# Intracranial Gunshot Wounds: An Assessment of Patient Characteristics on Surgical Outcomes

**DOI:** 10.7759/cureus.75412

**Published:** 2024-12-09

**Authors:** Yi-Ren Chen, Eli Johnson, Beatrice Ugiliweneza, Lily H Kim, Katie Shpanskaya, Maxwell Boakye, Victor Tse

**Affiliations:** 1 Department of Neurosurgery, Stanford University Medical Center, Stanford, USA; 2 Department of Neurosurgery, Duke University School of Medicine, Durham, USA; 3 Department of Health Management and Systems Science, University of Louisville, Louisville, USA; 4 Department of Neurosurgery, Stanford University School of Medicine, Stanford, USA; 5 Department of Radiology, Duke University School of Medicine, Durham, USA; 6 Department of Neurosurgery, University of Louisville School of Medicine, Louisville, USA; 7 Department of Neurosurgery, Kaiser Permanente, Redwood City, USA

**Keywords:** cranial trauma, gsw complications, gun shot wound, penetrating head wound, traumatic brain injury

## Abstract

Background/objective: Intracranial gunshot wounds (GSW) are often fatal, with most patients dying before intervention can occur. Surgical management, when indicated, results in decreased mortality. We sought to assess the neurosurgical outcomes and economic costs of intracranial GSW.

Methods: We conducted a retrospective analysis using the longitudinal claims Truven MarketScan® database (IBM) from 2000 to 2016. Mortality was the primary outcome of interest. Complications, length of stay, and payment were secondary outcomes. Multivariable logistic and linear regression analyses were performed to assess the relationship between age, gender, insurance type, and the number of comorbidities in the outcomes measured.

Results: We identified 315 patients (median age 34.0 years; interquartile range (IQR)=24, 48; 32.06% female) who received craniotomy or craniectomy for intracranial GSW. Mortality occurred in 44 patients (13.97%) and 234 patients (74.29%) experienced complications. The median length of stay was 13 days (IQR=5, 25 days), and the median cost was $70,624.00 (IQR=$32,378.00; $163,437.00). Increases in the Elixhauser index by one-comorbidity increments were associated with increased length of stay (risk ratio (RR)=1.207; 95% confidence interval (CI)=1.106-1.317) and payment (RR=1.135; 95% CI=1.036-1.243). Patients with respiratory complications, excluding infection, experienced an increased risk of mortality (odds ratio (OR)=3.486; 95% CI=1.623-7.485), length of stay (RR=1.649; 95% CI=1.321-2.060), and payment (RR=2.085; 95% CI=1.652-2.631).

Conclusions: Although these findings must be interpreted in the context of the limitations inherent to studies using national administrative data, the current study provides additional insight into the relationship between patient characteristics and outcomes after surgery for intracranial GSW.

## Introduction

The age-adjusted mortality rate from gunshot wounds (GSWs) has increased in the United States from 10.11 deaths per 100,000 people in 2000 to 11.73 deaths per 100,000 people in 2016 [1]. While injuries from GSWs can affect various parts of the body, those involving the brain are particularly devastating. Intracranial ballistic injuries are notably fatal with mortality rates ranging from 50%-90% [2,3]. More than half of the patients die before reaching the hospital, and the mortality rate for those who reach the emergency department has been reported to range between 50 and 90% depending on the study [2,4-6].

Treatment options include medical management, surgical intervention, or in select cases, endovascular therapy to address vascular injury [7-9]. When surgery is indicated, survival rates have increased with the use of aggressive operative management and intracranial monitoring [10]. These interventions are particularly relevant in cases of diffuse cerebral injury or increased intracranial pressure, where decompressive techniques may alleviate the risk of fatal brainstem herniation [11,12]. However, such interventions are not without risk. Intracranial GSWs represent open, contaminated wounds with a high potential for infection. Delays in treatment increase the likelihood of infectious complications, including abscess formation and meningitis, making timely, targeted intervention critical [13].

Despite the significant morbidity and mortality associated with intracranial GSWs, our understanding of factors that influence patient outcomes remains limited. In particular, penetrating brain injuries are relatively rare compared to other traumatic brain injuries, limiting the scope and scale of research dedicated to their management. Existing studies often rely on small cohorts or case series, which may not capture the broader spectrum of patient characteristics and outcomes [14].

In the present study, we use data from the MarketScan national administrative database (IBM) to examine the effect of patient characteristics on outcomes following surgical intervention for intracranial GSW in a more comprehensive manner. Specifically, we investigate how patient characteristics like age, gender, insurance status, and comorbidity burden correlate with outcomes, including survival rates and postoperative complications.

This article was previously presented as a meeting abstract at the 2019 AANS Annual Scientific Meeting (April 13-17, 2019), San Diego.

## Materials and methods

Data source

We queried an observational administrative database of patients in the United States who underwent craniotomy or craniectomy for penetrating firearm injury between 2000 and 2016. Utilization data in the inpatient setting were obtained from the Truven MarketScan® Commercial Claims and Encounters and the Medicare Supplemental and Coordination of Benefits databases (IBM). These data encompass healthcare claims submitted on behalf of individuals enrolled in private insurance plans and Medicare/Medicaid through a participating employer, health plan, or government organization. The data includes information on diagnoses, date of service, and patient demographics [15,16]. Institutional review board (IRB) approval from our institution was not required for this study.

Patient selection

We developed the patient cohort by utilizing Common Procedural Terminology (CPT) and International Classification of Diseases (ICD) codes to identify patients over 18 years of age who underwent craniectomy or craniotomy due to firearm injury between 2000 and 2016. For a full list of codes used to define our cranial firearm injury cohort, see Table 1.

**Table 1:** Search criteria for intracranial and firearms-related injuries and procedures ICD, International Classification of Diseases; CPT, Current Procedural Terminology

Category	ICD-9 Code	ICD-10 Code	Description
Intracranial Injuries			
	800.xx-804.xx	S06.xxxx	-
	850.xx-854.xx	-	-
Firearms	-	-	-
	E922	X93 - X95	-
	E955	W32 - W34	-
	E965	X72 - X74	-
	E970	Y23, Y24	-
	E985	Y350, Y360	-
	E991	Y374, Y384	-
CPT Codes	-	-	-
	61570	-	Craniectomy or craniotomy; with excision of foreign body from brain
	61571	-	Craniectomy or craniotomy; with treatment of penetrating wound of brain

Our final sample was 315 patients. Although the prevalence of intracranial GSWs from 2000 to 2016 is higher than 315, many victims never receive surgical intervention due to its very high mortality rate at the scene of the incident [17].

Explanatory variables

Explanatory variables were patient age, gender, insurance type, and extent of comorbidities. The burden of comorbidities was represented by the Elixhauser score, which was calculated using an adaptation to ICD-9 and ICD-10 codes developed by Quan et al. [18] and other studies [19-20].

Outcomes

The primary measure used to assess patient outcomes at the time of surgery was mortality. Secondary outcome measures were complications, length of stay, and costs. Complications were recorded if they occurred at the time of surgery or during hospital stay following the surgical procedure. Payment in this study includes the cost at the time of surgery and the length of hospital stay following the surgery after excluding any negative or zero-valued payments.

Statistical analysis

Data were presented using summary statistics. Continuous variables were summarized with mean, associated standard deviation, median, and interquartile range, as well as extremes (minimum and maximum). Categorical variables were summarized with count and percentage. Continuous patient characteristics and demographics were compared with Wilcoxon rank sum tests and categorical patient variables via Chi-square tests. Multivariable analysis was also performed using complications, mortality, length of stay, and payment as the testing variables while controlling for age, gender, insurance type, and Elixhauser score. All tests were two-sided, and a p-value of less than 0.05 was considered significant. All data pre-processing and analysis were performed in SAS 9.4 software (SAS Institute, Cary, NC).

## Results

Sample characteristics

We identified 315 patients (median age 34 years; interquartile range (IQR)= 24, 48) who received craniotomy or craniectomy between July 1, 2000, and December 31, 2016 (Table 2).

**Table 2:** Baseline patient demographical characteristics SD, Standard deviation; IQR, interquartile range.

Variables		Combined cohort (N=315)
Age (Years)		
	Mean (SD)	37.1 (15.5)
	Median (IQR)	34 (24, 48)
	Range	18-89
Female, n (%)		101 (32.06%)
Insurance		
	Commercial, n (%)	173 (54.92%)
	Medicaid, n (%)	127 (40.32%)
	Medicare, n (%)	15 (4.76%)
Elixhauser index		
	Mean (SD)	1.6 (1.3)
	Median (IQR)	1 (1, 3)
	Range=	0-5

The average age of the patients was 37.1 years (SD=15.5). There were 101 female patients (32.06%). In the final cohort, 173 patients had commercial insurance (54.92%), 127 had Medicaid (40.32%), while 15 had Medicare insurance (4.76%). The median Elixhauser index score was 1 (IQR=1,3; Range=0-5).

Complications and comorbidities

Of the 315 patients undergoing surgery for cranial wounds due to firearm injury, 234 (74.29%) experienced at least one complication at the time of or following surgery (Table 3).

**Table 3:** Complications and comorbidities

Complications	Total
	N=315
Respiratory, n (%)	185 (58.73%)
Cerebrovascular disease, n (%)	77 (24.44%)
Pneumonia, n (%)	38 (12.06%)
Vein thrombosis, n (%)	22 (6.98%)
Infection, n (%)	15 (4.76%)
Hydrocephalus, n (%)	12 (3.81%)
Renal, n (%)	9 (2.86%)
Nervous system complication, n (%)	3 (0.95%)
Cerebrospinal fluid rhinorrhea, n (%)	2 (0.63%)
Myocardial infarction, n (%)	2 (0.63%)
Wound, n (%)	1 (0.32%)
Any one of above, n (%)	234 (74.29%)
Comorbidities (Top 10)	Total
	N=315
Fluid and electrolyte disorders, n (%)	84 (26.67%)
Depression, n (%)	60 (19.05%)
Other neurological disorders, n (%)	54 (17.14%)
Paralysis, n (%)	49 (15.56%)
Weight loss, n (%)	40 (12.7%)
Cardiac arrhythmia, n (%)	36 (11.43%)
Hypertension, uncomplicated, n (%)	34 (10.79%)
Alcohol abuse, n (%)	28 (8.89%)
Drug abuse, n (%)	23 (7.3%)
Coagulopathy, n (%)	21 (6.67%)

The top three complications were respiratory failure (n=185, 58.73%), cerebrovascular disease (n=77, 22.44%) and pneumonia (n=38, 12.06%). The top three comorbidities were fluid and electrolyte disorders (n=86, 26.67%), depression (n=60, 19.05%), and other neurological disorders (n=54, 17.14%).

Outcomes

Among 315 patients, a significant majority experienced complications, with 234 patients (74.29%) having at least one complication during their hospital stay. The mortality rate for this cohort was 13.97%. The length of stay varied considerably among patients, with a mean duration of 20.1 days (SD = 27.8 days). The financial cost of treatment, measured in 2016 dollars, also showed a wide range. The mean payment per patient was $130,534 (SD = $163,273), while the median payment was $70,624, with an IQR of $32,378 to $163,437. Payments ranged from $0 to a maximum of $1,352,678, illustrating the substantial economic burden for patients with prolonged hospitalizations or multiple interventions (Table 4).

**Table 4:** Outcomes SD, Standard deviation; IQR, interquartile range.

Variables		Combined Cohort (N=315)
Complications, n (%)		234 (74.29%)
Mortality, n (%)		44 (13.97%)
Length of stay (days)		
	Mean (SD)	20.1 (27.8)
	Median (IQR)	13 (5, 25)
	Range, min-max	1-337
Payment ($ in 2016)		
	Mean (SD)	130534 (163273)
	Median (IQR)	70624 (32378, 163437)
	Range, min-max	0-1352678

Adjusted effect of patient characteristics on outcomes

The primary and secondary outcomes varied as we accounted for age, gender, type of insurance, and Elixhauser index (Table 5).

**Table 5:** The adjusted effect of patient characteristics on outcomes OR, odds ratio; CI, confidence interval; RR, risk ratio.

		Mortality	Length of stay	Payment	Complications
Variables		OR (95% CI)	RR (95% CI)	RR (95% CI)	OR (95% CI)
Age, 1-year increment		1.011 (0.985 - 1.039)	0.998 (0.989 - 1.007)	1.004 (0.995 - 1.013)	1.007 (0.986 - 1.028)
Gender: female vs male		0.99 (0.482 - 2.032)	0.956 (0.755 - 1.212)	0.979 (0.764 - 1.256)	0.637 (0.373 - 1.09)
Insurance	Medicaid vs commercial	0.91 (0.434 - 1.91)	1.194 (0.948 - 1.503)	0.509 (0.4 - 0.649)	0.884 (0.515 - 1.518)
	Medicare vs commercial	1.361 (0.271 - 6.848)	0.478 (0.26 - 0.881)	0.155 (0.082 - 0.292)	0.935 (0.202 - 4.327)
Elixhauser Index, 1 increment		0.827 (0.623 - 1.097)	1.207 (1.106 - 1.317)	1.135 (1.036 - 1.243)	1.092 (0.886 - 1.346)
Respiratory, yes vs no		3.486 (1.623 - 7.485)	1.649 (1.321 - 2.06)	2.085 (1.652 - 2.631)	-
Cerebrovascular Disease, yes vs no		1.079 (0.491 - 2.373)	1.252 (0.967 - 1.619)	1.242 (0.948 - 1.627)	-
Pneumonia, yes vs no		0.123 (0.016- 0.945)	1.997 (1.425 - 2.799)	1.797 (1.264 - 2.556)	-
Vein thrombosis, yes vs no		0.275 (0.034- 2.222)	1.942 (1.253 - 3.008)	2.426 (1.531 - 3.845)	-

Increasing age by one-year increments demonstrated no difference in complications (odds ratio (OR)=1.007; 95% confidence interval (CI)=0.986 - 1.028), mortality (OR=1.011; 95% CI=0.985 - 1.039), length of stay (risk ratio (RR)=0.998; 95% CI=0.989 - 1.007), and payment (RR=1.004; 95% CI= 0.995 - 1.013). There were no significant effects of gender on complications (OR=0.637; 95% CI=0.373 - 1.090), mortality (OR=0.99; 95% CI=0.482 - 2.032), length of stay (RR=0.956; 95% CI=0.755 - 1.212), or payments (RR=0.979; 95% CI=0.764 - 1.256). When comparing Medicaid to commercial insurance, patients with Medicaid had less total payment (RR=0.509; 95% CI=0.400 - 0.649). Medicare compared to commercial insurances revealed that patients with Medicare had a shorter length of stay (RR=0.478; 95% CI=0.260 - 0.881) and less payment (RR=0.155; 95% CI=0.082 - 0.292). Finally, increases in the Elixhauser index by one-comorbidity increments were associated with longer length of stay (RR=1.207; 95% CI=1.106 - 1.317) and higher payment (RR=1.135; 95% CI=1.036 - 1.243).

Patients with a respiratory complication experienced an increased risk of mortality (OR=3.486; 95% CI=1.623 - 7.485), length of stay (RR=1.649; 95% CI=1.321 - 2.06), and payment (RR=2.085; 95% CI=1.652 - 2.631). The presence of cerebrovascular disease was not associated with mortality (OR=1.079; 95% CI=0.491 - 2.373), length of stay (RR=1.252; 95% CI=0.967 -1.619), and payment (RR=1.242; 95% CI=0.948 - 1.627). When analyzing patients with pneumonia, there was decreased association with mortality (OR=0.123; 95% CI=0.016 - 0.945) and payment (RR=1.797; 95% CI=1.264 - 2.556) as well as increased length of stay (RR=1.997; 95% CI=1.425 - 2.799). Those with vein thrombosis were associated with increased length of stay (RR=1.942; 95% CI=1.253 - 3.008) and payment (RR=2.426; 95% CI = 1.531 - 3.845).

## Discussion

In the present study, we sought to examine the outcomes of penetrating cranial gunshot wounds immediately after surgery. We report that Medicaid versus commercial insurance was associated with an increased length of stay and decreased cost. In addition, a higher comorbidity burden was associated with increased length of stay and cost. The presence of respiratory complications was linked to increased rates of mortality, length of stay, and cost. In our cohort, age and gender differences did not play a significant role in the outcomes measured.

Mortality in intracranial ballistic injury from firearms is high due to traumatic damage inflicted on the brain. As the bullet penetrates the cranium, damage occurs from direct crushing injury, shockwaves, and transient cavitation [21]. Aras et al. reported a significantly decreased mortality with the use of surgical management (p<0.001), with mortality in 19.32% of patients (n=17/88) receiving surgery and 42.86% of patients (n=42/98) receiving conservative management [22]. In our cohort, 13.97% of patients (n=44/315) died during operative management or in their stay following surgery. Among the known predictors of mortality in intracranial GSW, such as age, pupillary responses, and bihemispheric injuries, the initial Glasgow Coma Scale (GCS) is considered the strongest predictor [14,23]. Unfortunately, our analysis of mortality in the cohort is limited by an inability to obtain GCS from the MarketScan database (IBM).

Complications are a significant risk in all surgical interventions. In the present study, 234 patients (74.29%) experienced one or more complications. The top five complications were respiratory failure, cerebrovascular disease, pneumonia, vein thrombosis, and infection. Previous studies have found that the risk of infection is higher in cases where bone fragments have not been completely removed [13,22,24]. Other common complications include CSF rhinorrhea and leaks, brain abscesses, intracranial aneurysms, and post-traumatic epilepsy [2,17,22]. In the present study, respiratory complications were particularly devastating and associated with increased mortality (OR=3.486; 95% CI=1.623 - 7.485). In the setting of trauma, ventilator-associated pneumonia (VAP) is a common complication, particularly in patients with head and neck trauma [25-27]. It is unclear whether VAP increases the risk of death following traumatic brain injury [27,28]. Interestingly, the presence of pneumonia was protective against death (OR=0.123; 95% CI=0.016 - 0.945). These complications play an essential role in surgical planning and post-operative recovery and, as such, should be considered in clinical decision-making. 

As with all major surgeries of the brain, costs are high, given the complexity of the central nervous system and intensive postsurgical management. We found that the median length of stay was 13 days (IQR: 5 - 25 days), and the median cost was $70,624.00 (IQR: $32,378.00 - $143,437.00). Pereira et al., in a study of 702 patients who sustained facial and intracranial GSW, reported length of stay that ranged from seven to 14 days, with half of the patients requiring intensive care and an average cost of $101,903 [29]. In our cohort, there were 127 patients with Medicare (40.32%), and this insurance type was associated with less payment (RR=0.571; 95% CI=0.454 - 0.718). Additionally, patients with an increased number of complications, as evidenced by the Elixhauser index, incurred a longer length of stay and payments. In particular, the presence of respiratory complications, pneumonia, and deep vein thrombosis were all linked to increased length of stay and payment.

Previous research has implicated age as an important contributor to increased complications and mortality rates in traumatic brain injury [30]. However, studies specifically looking at advanced age and outcomes in intracranial GSW are less conclusive [1,13]. Our results show that increasing age was not associated with higher rates of complications (OR=1.007; 95% CI=0.986 - 1.028), mortality (OR=1.011; 95% CI=0.985 - 1.039), length of stay (RR=0.998; 95% CI=0.989 - 1.007) and payment (RR=1.004; 95% CI= 0.995 - 1.013). In a study by Holmes et al. consisting of 677 trauma patients over the age of 55, an increased number of comorbidities was associated with poor outcomes [31]. An increased number of comorbidities, as evidenced by the Elixhauser index in the current study, was associated with longer length of stay (RR=1.207; 95% CI=1.106 - 1.317) and higher payment (RR=1.135; 95% CI=1.036 - 1.243). However, there was no associated increase in the mortalities or complications.

Limitations

The results of the present study should be interpreted considering the limitations inherent to large database studies. Specifically, our reporting of cranial gunshot wounds, complications, comorbidities, and outcomes assumes the accuracy of the administrative data. Our study lacks several additional details regarding the injury, such as the site of the wound or other involved organs, surgical management (extent of craniotomy or craniectomy) as well as data on the level of care during hospitalization (i.e., intensive care versus ward). The incidence of cranial gunshot wounds based on CPT, ICD-9, and ICD-10 codes may underestimate the actual prevalence. Although we found that Medicare was associated with decreased length of stay and lower cost, our sample size for this group was too small (n=15) compared to our commercial insurance groups (n=173). Further work will be required to investigate this relationship and its effects on patient outcomes.

## Conclusions

Overall, the current study provides insight into the link between patient age, insurance type, and comorbidities, with outcomes after surgery for intracranial GSW. The study highlights significant morbidity and morbidity associated with intracranial gunshot wounds as well as the complexity of surgically managing these patients. Patient factors, such as the number of comorbidities and the presence of respiratory complications, are strongly associated with increased length of stay, costs, and risk of mortality. Further work may identify the long-term effects of these characteristics on surgical outcomes and assist with better-informed clinical decisions.
